# Endophytic Fungi from *Lycium chinense* Mill and Characterization of Two New Korean Records of *Colletotrichum*

**DOI:** 10.3390/ijms150915272

**Published:** 2014-08-28

**Authors:** Narayan Chandra Paul, Hyang Burm Lee, Ji Hye Lee, Kyu Seop Shin, Tae Hee Ryu, Hye Ri Kwon, Yeong Kuk Kim, Young Nam Youn, Seung Hun Yu

**Affiliations:** 1 Division of Applied Bioscience & Biotechnology, College of Agriculture & Life Sciences, Chonnam National University, Gwangju 500-757, Korea; E-Mails: ncpaul@jnu.ac.kr (N.C.P.); hblee@jnu.ac.kr (H.B.L.); 2 Department of Agricultural Biology, College of Agriculture & Life Sciences, Chungnam National University, Daejeon 305-764, Korea; E-Mails: leejh224@nate.com (J.H.L.); seopbee@korea.kr (K.S.S.); fbxogml89@naver.com (T.H.R.); hr7345@nate.com (H.R.K.); 3 Department of Herbal Crop Research, National Institute of Horticultural & Herbal Science, Rural Development Administration (RDA), Eumseung 369-873, Korea; E-Mail: kimyguk@korea.kr

**Keywords:** colletotrichum brevisporum, colletotrichum fructicola, endophytic fungi, lycium chinense mill, new records

## Abstract

Chinese boxthorn or matrimony vine (*Lycium chinense* Mill) is found primarily in southeastern Europe and Asia, including Korea. The dried ripe fruits are commonly used as oriental medicinal purposes. Endophytic fungi were isolated from surface sterilized tissues and fruits of the medicinal plant in 2013 to identify the new or unreported species in Korea. Among 14 isolates, 10 morphospecies were selected for molecular identification with the *internal transcribed spacer* (*ITS*) gene. Phylogenetic analysis revealed that all isolates belonged to Ascomycota including the genera *Acremonium*, *Colletotrichum*, *Cochliobolus*, *Fusarium*, *Hypocrea* and *Nemania*. Two *Colletotrichum* species were identified at the species level, using three genes including *internal transcribed spacer* (*ITS*), glycerol-3-phosphate dehydrogenase (GAPDH) and Actin (ACT) for PCR and molecular data analysis along with morphological observations. The fungal isolates, CNU122031 and CNU122032 were identified as *Colletotrichum fructicola* and *C. brevisporum*, respectively. Morphological observations also well supported the molecular identification. *C. brevisporum* is represented unrecorded species in Korea and *C. fructicola* is the first record from the host plant.

## 1. Introduction

Endophytes are microorganisms that reside within internal tissues of living plants without visibly harming the host plant [1]. Endophytic microorganisms have been found in all plant families [2], including species in many different climate regions of the world [2,3,4,5]. Endophyte research has increased in recent years because of their taxonomic diversity [6], their multiple functions including potential uses as genetic vectors [7], and their influence on host plant growth promotion and fitness [8,9,10]. Recently, there has been a rising interest in the use of eco-friendly biopesticides for control of plant diseases, especially for biological control of plant diseases, and has few quick benefits but can be long lasting, inexpensive, and harmless. Bio-control systems do not eliminate neither pathogen nor disease but bring them into natural balance. Endophytes are a major source of biological control agents these days.

Chinese boxthorn (*Lycium chinense* Mill) is also known as matrimony vine, goji berry or wolfberry and belongs to the Solanaceae family. The plant is native to southeastern Europe and Asia. The fruits are well known in China and Korea for their medicinal and high nutritious value [11]. Consumers of Chinese boxthorn are increasing dramatically, mainly due to the nutrition value (68% of the dry mass exists as carbohydrates, 12% as protein, 10% as fiber and 10% as fat) and there is also high proportion of antioxidants [12]. The plant may live in association with a number of endophytes. Researchers have attempted to isolate fungi from this plant in Korea.

*Colletotrichum* causes anthracnose diseases in a wide range of economically important plants, crops, and grasses and is important fungal taxa. More than one species of *Colletotrichum* can affect a single plant species [13]. This phenomenon makes the taxa more important in agriculture. Identification based on morphology is problematic due to the small number of morphological traits that can be used to distinguish species [14]. Conidial size, shape, appresoria formation, sclerotia, setae, and acervuli are some of their distinguishing characters used to separate species of *Colletotrichum* [15,16]. Recently, multilocus molecular characteristics have become increasingly important in the identification of *Colletotrichum* species [17].

The objectives of this study were (1) to investigate the occurrence, isolation and sequence based identification of endophytic fungi from symptomless tissues of Chinese boxthorn plant in Korea and (2) to isolate, identify, and characterize two new *Colletotrichum* species by molecular and morphological data analysis.

## 2. Results

A total of 10 endophytic fungal morphospecies obtained from *L. chinense* in Korea were selected from 14 isolates for identification (Table 1). Endophytic fungi were identified by analysis of the ITS region of the rDNA gene.

**Table 1:** Closest relatives of endophytic fungi isolated from *Lycium chinense* with BLAST search analyses based on *ITS* gene sequence.

Isolate No.	GenBank Closest Hit (Accession Number)	Similarity (%)	Sequence Based Identification	Host Tissue	Accssion Number
CNU122031	*C. fructicola* C1263.3 (JX010164)	100	*C. fructicola*	Fruit	KJ651254
CNU122032	*C. brevisporum* LC0600 (KC790943)	99	*C. brevisporum*	Leaf	KJ651255
	*Glomerella magna* L2.5 (DQ003103)	99			
CNU122033	*Acremonium* sp. r116 (HQ649797)	100	*Acremonium* sp.	Fruit	KJ651256
	*Acremonium strictum* F21 (EU497953)	99			
CNU122034	*Cochliobolus lunatus* Cs-1C (JN107740)	99	*Cochliobolus lunatus*	Leaf	KJ651257
	*Cochliobolus* sp. P2E4 (JN207244)	99			
CNU122035	*Fusarium* cf. *equiseti* AM-48 (JN038489)	99	*Fusarium equiseti*	Leaf	KJ651258
	*Fusarium equiseti* ATT040 (HQ607811)	99			
CNU122036	*C. truncatum* tc-1 (KC460308)	99	*Colletotrichum* sp.	Leaf	KJ651259
	*Colletotrichum* sp. ITCC 2041 (JN390888)	99			
CNU122037	*Hypocrea citrina* GJS 91-61 (DQ000630)	99	*Hypocrea citrina*	Fruit	KJ651260
CNU122038	*Nemania* sp. NDJL-2009a (GU166482)	99	*Nemania* sp.	Leaf	KJ651261
	*Nemania serpens* BF330 (EF155504)	95			
CNU122039	*Nemania* sp. AX48 (KC507255)	100	*Nemania* sp.	Leaf	KJ651262
	*Nemania diffusa* Z26 (JN198514)	100			
CNU122040	*Nemania* sp. AX48 (KC507255)	99	*Nemania* sp.	Leaf	KJ651263

**Figure 1:**
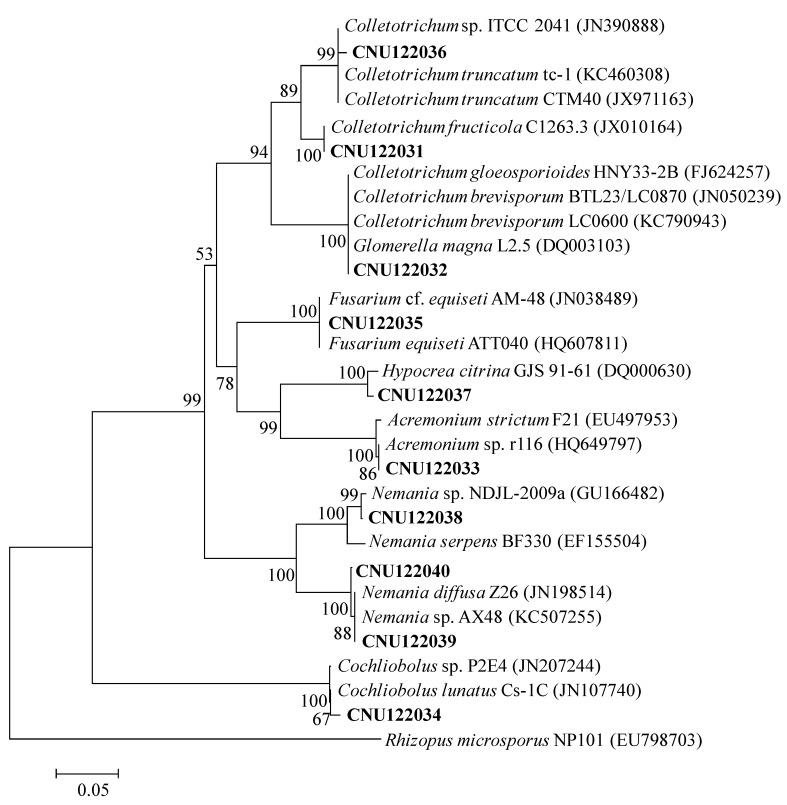
Neighbor-joining phylogenetic tree showing the placement of the representative endophytic isolates based on the sequences of the ITS region. The Kimura two-parameter model is used for pairwise distance measurement. The tree is rooted with *Rhizopus microsporus* (EU798703)*.* Only bootstrap values *>*50% (1000 replications) are shown in at the internal nodes.

Six distinctive fungal taxa were detected at a *>*90% sequence similarity threshold (Table 1) representing six fungal taxa (Figure 1) and they were *Acremonium*, *Cochliobolus*, *Colletotrichum*, *Fusarium*, *Hypocrea* and *Nemania*. The species of fungi isolated from Chinese boxthorn were *Acremonium* sp. (CNU122033), *Cochliobolus lunatus* (CNU122034), *Colletotrichum brevisporum* (CNU122032), *Colletotrichum fructicola* (CNU122031), *Colletotrichum* sp. (CNU122036), *Fusarium equiseti* (CNU122035), *Hypocrea citrina* (CNU122037), and *Nemania* sp. (CNU122038, CNU122039, & CNU122040).

The sequence of CNU122031 completely matched with *C. fructicola* C1263.3 when retrieved from GenBank with maximum bootstrap value shown in the phylogenetic tree. The isolate CNU122032 showed 99% sequence similarity with two similar isolates *C. brevisporum* LC0600 and *Glomerella magna* L2.5 with 100% bootstrap support. Therefore, we used Actin and GAPDH primers to confirm the identification, and sequence similarity with *C. brevisporum* was 99%–100%. The isolate CNU122033, CNU122034, CNU122035, CNU122036 and CNU122037 showed 99%–100% sequence similarity with *Acremonium* sp. r116, *Cochliobolus lunatus* Cs-1C, *Fusarium* cf. *equiseti* AM-48, *Colletotrichum* sp. ITCC 2041 and *Hypocrea citrina* GJS 91–61, and a high bootstrap value. CNU122038, CNU122039 and CNU122040 showed 99%–100% sequence similarity with *Nemania* species isolates from GenBank.

### 2.1. Taxonomy of Two Colletotrichum Species

#### 2.1.1. Molecular Phylogeny

**Figure 2:**
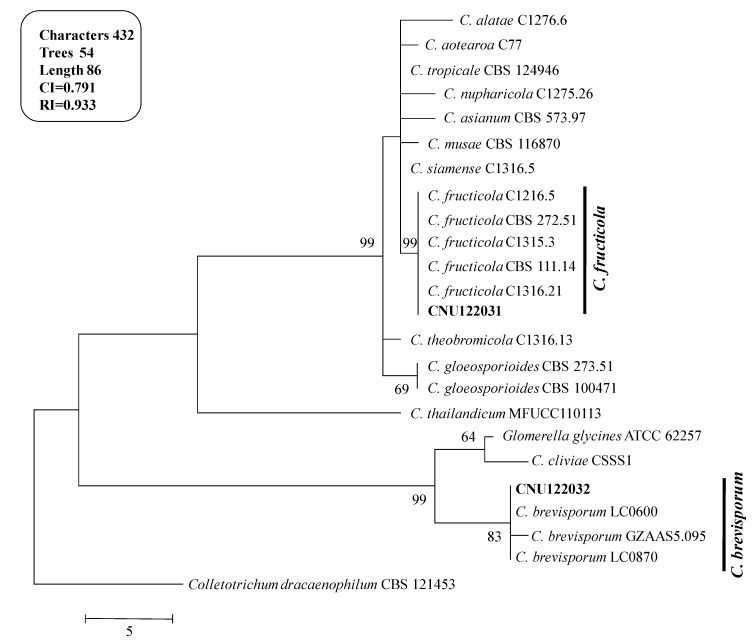
Phylogenetic tree showing the placement of the *Colletotrichum* isolates from the present study and their related species generated using the maximum parsimony analysis of combined dataset of *ITS*, ACT, and GAPDH gene sequences. Numbers at the nodes indicate bootstrap values (*>*50%) from 1000 replications. The bar indicates the number of substitutions per position. The tree is rooted with *C. dracaenophilum* CBS121453.

A molecular phylogenetic analysis was generated using the multilocus molecular dataset (Figure 2). The combined dataset of *ITS*, ACT and GAPDH comprised 24 sequences and produced 54 most parsinimonious trees (TL = 86, CI = 0.791 and RI = 0.933). One of the most parsimonious trees is shown in Figure 2.

The isolates CNU122031 and CNU122032 formed a monophyletic clade with reliable reference relatives from GenBank isolates. The phylogenetic tree constructed from the combined dataset of ITS, GAPDH, and ACT gene sequences clearly showed that the isolates CNU122031 and CNU122032 are *C. fructicola* and *C. brevisporum*, respectively with high bootstrap support (Figure 2).

#### 2.1.2. Morphological Characterization

Taxonomic descriptions and micromorphographs of the morphological structures for the two species (*C. fructicola* CNU122031 and *C. brevisporum* CNU122032) are shown in details below.

#### 2.1.3. CNU122031-*Colletotrichum fructicola*

**Figure 3:**
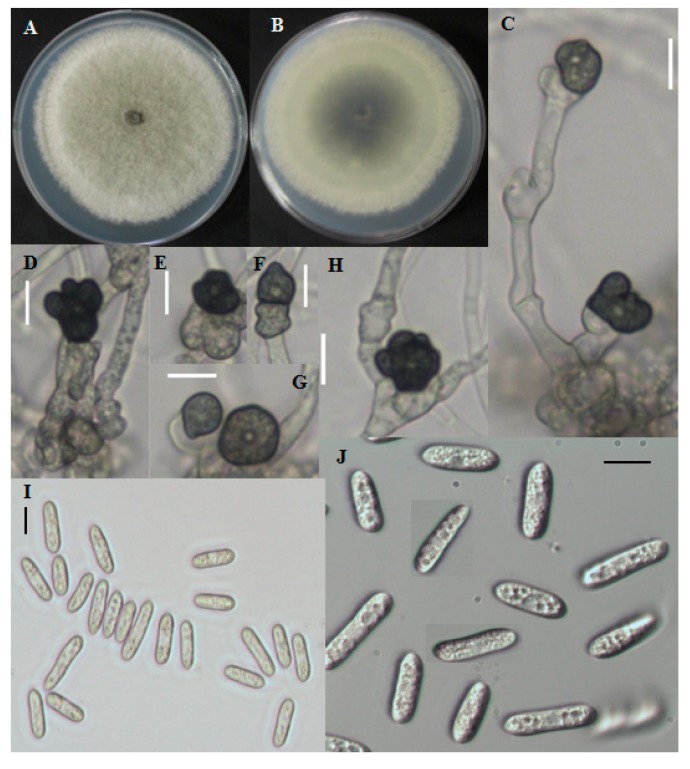
Morphological features of *Colletotrichum fructicola* CNU122031. Colony morphology in PDA after 7 days of inoculation at 25 °C (**A**, obverse; **B**, Reverse); **C**–**H**: appresoria; **I**,**J**: Conidia (scale bars **C**–**J** = 10 μm).

Fast growing colonies on PDA were white at the starting of the growth period. Over time, colonies became gray to dark gray at the center (Figure 3A,B). The reverse color was grayish to blackish with a white halo. Aerial mycelia were whitish, dense, cottony, and without visible conidial masses. Single-celled conidia were observed after the sporulation period. The cylindrical conidia had obtuse to rounded ends and the size ranged from 8.7–29.5 μm long and 2.8–5.9 μm wide (Figure 3I,J). The shape of appresoria varied among species. Appresoria were commonly ovoid, sometimes clavate. The sizes of the appresoria were 10.5–14.5 × 6–11 μm (Figure 3C–H).

#### 2.1.4. Distinguishing Characters

*C. fructicola* CNU122031 was distinctly separated from the closely related species *C. siamense* by colony color, conidial size, and shape. *C. siamense* produced a pinkish colony color and the reverse was yellowish to pinkish whereas, the present isolate produced gray to dark gray colony color, and completely matched with the reference species, *C. fructicola* strains [18]. The size of the colony of present isolate was shorter than that of *C. siamense*. The CNU122031 isolate produced cylindrical, obtuse conidia but *C. siamense* produced fusiform conidia, indicating a clear difference between the species (Table 2). The fungus, *C. fructicola* is a newly isolated species from *L. chinense*.

**Table 2:** Morphological characters of *Colletotrichum* species described in this study and closely related reference species.

Taxa	Colony	Conidia Shape and Size (μm)	Appresoria Size (μm)	Reference
*C. brevisporum*	White, mycelium in small tufts, reverse dark in middle	Cylindrical with round ends. 12.2–24.2 × 2.6–6	10–16.8 × 5–9.4	This study
*C. brevisporum*	Aerial mycelia in small tufts, white, sparse with conidial masses, reverse dark green	Cylindrical with round ends, smooth-walled, hyaline. 12–17 × 5–6	10.5–14.5 × 8−11	[13]
*C. cliviae*	White to gray, white at margin, reverse dark brown to greenish black	Cylindrical, straight or slightly curved, obtuse at the ends. 19.5–24.5 × 4.5–7	10.5–14.5 × 6−11	[19]
*C. fructicola*	White, becoming gray to dark gray at the centre with age, dark circular margin at the center in reverse	Cylindrical with obtuse to rounded ends. 8.7–29.5 × 2.8–5.9	4.1–5.4 × 3−4.9	This study
*C. fructicola*	White, becoming gray to dark gray at the centre with age, dark circular around the growing margin at the center in reverse	Cylindrical with obtuse to slightly rounded ends, sometimes oblong, hyaline. 9.7–14 × 3–4.3	4.7–8.3 × 3.5−5	[18]
*C. siamense*	White, becoming pale brownish to pinkish, pale yellowish to pinkish colonies in reverse	Fusiform, sometimes with obtuse to slightly rounded ends, sometimes oblong, hyaline. 7–18.3 × 3–4.3	4.7–10.7 × 3.3–6.7	[18]

#### 2.1.5. CNU122032-*Colletotrichum bresvisporum*

**Figure 4:**
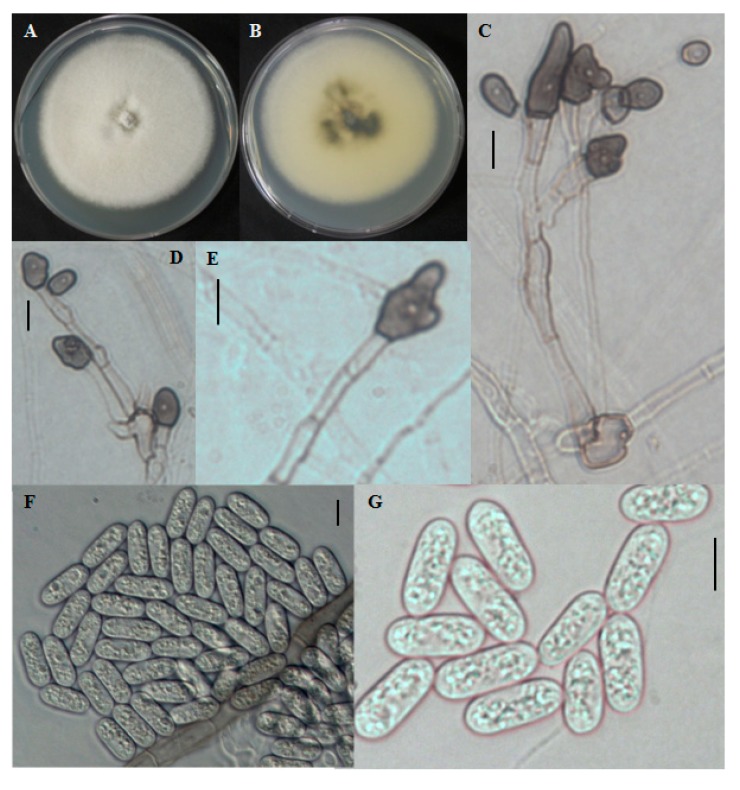
Morphological features of *Colletotrichum brevisporum* CNU122032. Colony in PDA after 7 days of inoculation at 25 °C (**A**, obverse; **B**, reverse); **C**–**E**: appresoria; **F**,**G**: Conidia (scale bars **C**–**G** = 10 μm).

The colony on PDA was white at first, became grayish over time. The mycelium was in small tufts. The reverse was dark blackish at the center and the edge was white to grayish with a white halo (Figure 4A,B). Aerial mycelia were white in color and dense. Conidial masses were not observed. Conidia produced were single celled. Conidia were cylindrical with rounded ends, smooth-walled, hyaline, and ranged in size from 12.2–24.2 × 2.6–6 μm (Figure 4F,G). Appresoria were 10–16.8 × 5–9.4 μm in slide cultures, irregular in shape, sometimes ovoid, often becoming complex over time (Figure 4C–E).

#### 2.1.6. Distinguishing Characters

The isolate was identified as *C. brevisporum* by molecular data analysis. The morphological characteristics supported the molecular identification.

The isolate was closely related to *C. cliviae*, but differed in conidial shape and length. Conidia of the present isolate were cylindrical with distinctly round ends, whereas the conidia of *C. cliviae* were cylindrical, straight, or curved, and obtuse at the ends. The present isolate did not produce any curved conidia, and not obtuse at the ends (Table 2). The morphological description of the present isolate completely matched that of *C. brevisporum* described by Noireung *et al*. 2012 [13]. This is the first fungal record of *C. brevisporum* isolated in Korea.

## 3. Discussion

Endophytic fungal distributions vary with plant-associated habitats which may affect microbial communities that colonize roots, leaves, stems, branches, fruits, pods, and leaves [20]. Previous investigations revealed that endophytes were isolated from different plant tissues or organs. In this study, we isolated and characterized endophytic fungi from leaves and fruits of the *L. chinense* Mill plant and identified two unreported fungi isolated from the *L. chinense* Mill plant.

Endophytic fungal communities associated with various kinds of plants from tropical, sub-tropical, temperate, and Arctic ecosystems were investigated previously and fungal diversity was high [21]. From the Chinese boxthorn plant we isolated 14 fungi. Among them, 10 morphospecies were selected and sequenced with *ITS* gene. Six different genera were observed (*Acremonium*, *Colletotrichum*, *Cochliobolus*, *Fusarium*, *Hypocrea* and *Nemania*) and included common endophytic fungi from different regions around the world. The distribution of dominant species agreed with previous report [6,7,22,23,24,25]. The Dominant fungi described here are commonly associated with disease symptoms in several crop plants. Species of *Acremonium*, *Colletotrichum*, *Cochliobolus*, *Fusarium*, *Hypocrea*, and *Nemania* cause severe disease in many cultivated and non-cultivated plants. The anthracnose of chili pepper caused by *C. acutatum* is an example and *Fusarium oxysporum* causes disease in the same plant and many other cultivated and non-cultivated plants, and both were found in endophytic association with leaves, roots, pods, and many other plants. They were found in association with leaves and stems of medicinal plant *Tylophora indica* in India [24], and with the orchid species, *Dendrobium loddigesii* in China [6]. We isolated fungi associated with a host plant that did not show any disease symptoms. It is possible that they are latent pathogens.

A number of endophytic fungi consist of sterile mycelia or non-sporulating fungi and consequently cannot be identified by morphological characters. Previously, molecular techniques have been employed successfully for the identification of different endophytic fungal community [6,21,26]. In this study, we also used the molecular strategy to identify fungi specifically utilizing of ITS rDNA gene sequence and phylogenetic analysis.

## 4. Experimental Section

### 4.1. Sampling

**Figure 5:**
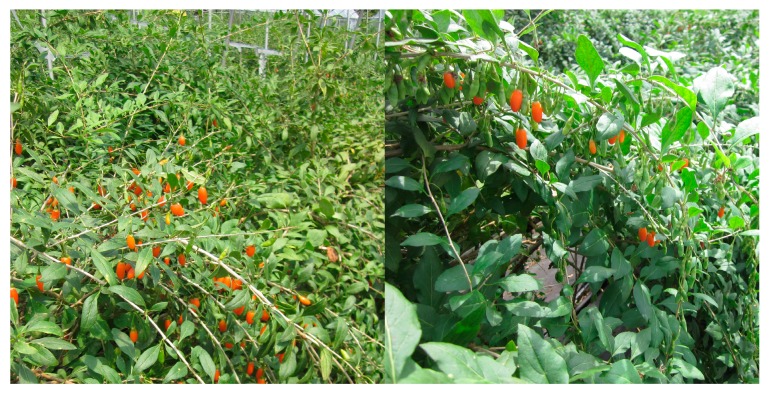
Chinese boxthorn (*Lycium chinense* Mill) plant tissues were collected from farmer’s field in Cheongyang locality in Korea.

Chinese boxthorn (*Lycium chinense* Mill) plants were selected because of their medicinal applications in Korea. Medicinal plants may live in association with a number of new or unreported microorganisms, especially fungal species. Sampling sites of this study were Cheongyang farmer’s field, South Chungcheong province, Republic of Korea (Figure 5). Living symptomless leaf and fruit samples were collected and stored in sterile polyethylene bags.

### 4.2. Isolation of Endophytic Fungi

Samples were cleaned under running tap water to remove debris, then air dried and processed within 5 h of collection. Tissues were cut into small (1 cm length and 0.5 cm width) pieces. Then surface sterilized by immersing in 95% ethanol for 1 min, sodium hypochlorite (4% chlorine) for 4 min and 95% ethanol for 30 s, and then samples were washed in sterile water three times to remove the surface sterilization agents. Samples were allowed to dry on paper towel in a laminar air flow chamber. A total of 120 segments were selected and plated (100 segments from leaf samples and 20 segments from fruit samples). Ten segments per petri dish were placed horizontally in potato dextrose agar (PDA, Difco, Franklin Lakes, NJ, USA) and rose bengal chloramphenicol agar (DRBC, Difco, Franklin Lakes, NJ, USA) supplemented with the antibiotic streptomycin sulfate (0.4 mg/mL, SIGMA-ALDRICH, Munich, Germany) to stop bacterial growth. After incubation at 25 °C for 5, 10, and 25 days, individual hyphal tips of the developing fungal colonies were collected and placed onto PDA media and incubated for 5–10 days, and checked for culture purity. Eventually, pure cultures were transferred to PDA slant tubes and eppendorf tubes with 20% glycerol stock solution. Strain numbers were assigned for selected isolates and deposited in the Fungal Herbarium of the Chungnam National University, Daejeon, Korea. Cultures of the isolates were also deposited in the Environmental Microbiology Lab. (EML) Herbarium, Chonnam National University, Gwangju, Korea.

### 4.3. Genomic DNA Extraction, PCR and Sequencing

Genomic DNA was extracted from 10 pure culture isolates using the method of Park *et al*. [27]. Primers ITS1 (5'-TCCGTAGGTGAACCTGCGG-3') and ITS4 (5'-TCCTCCGCTTATTGATATGC-3') were used for the amplification of the fungal rDNA internal transcribed spacers (ITS) regions of all isolates.

PCR amplification was carried out [28] in an i-Cycler (BIO-RAD, Hercules, CA, USA) for 30 cycles of 94 °C for 1 min denaturing, 55 °C for 1 min annealing and 72 °C for 1.30 min extension. Initial denaturing at 94 °C was extended to 5 min and the final extension was for 10 min at 72 °C. For the specific identification of the *Colletotrichum* species, two additional genes were used for PCR amplification: Actin (Forward ACT-512F & reverse ACT-783R) and Glyceraldehyde-3-phosphate dehydrogenase (Forward GDF & reverse GDR) (Table 3). The PCR conditions for these genes are showing in Table 3. All PCR products were purified using the Wizard PCR prep kit (Promega, Madison, WI, USA). Purified double-stranded PCR fragments were directly sequenced with BigDye terminator cycle sequencing kits (Applied Biosystems, Foster City, CA, USA) following the manufacturer instructions. The gel electrophoresis and data collection were performed on an ABI prism 310 Genetic Analyzer (Applied Biosystems, Foster City, CA, USA).

**Table 3:** Primers used in this study, with sequences and sources.

Gene	Product Name	Primer	Direction	Sequence (5'–3')	Reference
ACT	Actin	ACT-512F	Forward	ATGTGCAAGGCCGGTTTCGC	[29]
		ACT-783R	Reverse	TACGAGTCCTTCTGGCCCAT	[29]
GAPDH	Glyceraldehyde-3-phosphate dehydrogenase	GDF	Forward	GCCGTCAACGACCCCTTCATTGA	[30]
		GDR	Reverse	GGGTGGAGTCGTACTTGAGCATGT	[30]
*ITS*	*Internal transcribed spacer*	ITS-1F	Forward	CTTGGTCATTTAGAGGAAGTAA	[28]
		ITS-4	Reverse	TCCTCCGCTTATTGATATGC	[28]

### 4.4. Phylogenetic Analysis

The rDNA *ITS*, Actin, and GAPDH gene sequences were compared by BLAST search with sequence available in the GenBank database. Sequences generated from materials in this study and retrieved from GenBank (Table 4) were initially aligned using CLUSTAL X [31], and the alignment was refined manually using PHYDIT program version 3.2 [32]. A neighbor-joining tree for the ITS sequences was constructed with Kimura’s 2-parameter distance model [33] using the PHYLIP 3.57c package [34]. Maximum parsimony trees were constructed for the combined datasets of *ITS*, Actin, and GAPDH gene sequences using MEGA5 program. Bootstrap analysis using 1000 replications was performed to assess the relative stability of the branches.

**Table 4:** Isolates used in this study for molecular data analysis.

Species	Isolate	Host	Origin	Accession No.		
				*ITS*	gpd	ACT
***C. fructicola***	**CNU122031**	*Lycium chinense*	**Korea**	**KJ651254**	**KJ651266**	**KJ651264**
***C. brevisporum***	**CNU122032**	*Lycium chinense*	**Korea**	**KJ651255**	**KJ651267**	**KJ651265**
*C. alatae*	C1276.6	*Discorea alata*	India	JX010191	JX010011	JX009470
*C. aotearoa*	C77	*Vitex lucens*	NZ ^b^	JX010221	JX010023	JX009500
*C. asianum*	CBS 573.97	*Mangifera indica*	Brazil	KC566732	KC566586	KC566878
*C. brevisporum*	GZAAS5	*Citrus* sp.	China	JQ247623	JQ247599	JQ247647
*C. brevisporum*	LC0600	*Neoregalia* sp.	Thailand	JN050238	JN050227	JN050216
*C. brevisporum*	LC0870	*Pandanas pygmaeus*	Thailand	JN050239	JN050228	JN050217
*C. cliviae*	CSSS1	*Clivia miniata*	China	GQ485607	JX546611	GU085861
*C. dracaenophilum*	CBS 121453	*Dracaena sanderiana*	Bulgaria	EU003533	NA ^a^	NA ^a^
*C. fructicola*	C1316.21	*Theobroma cacao*	Panama	JX010173	JX009992	JX009581
*C. fructicola*	CBS 111.14	NA ^a^	Brazil	KC566785	KC566639	KC566931
*C. fructicola*	CBS 272.51	NA ^a^	Brazil	KC566783	KC566637	KC566929
*C. fructicola*	C1315.3	*Coffea arabica*	Thailand	JX010165	JX010033	JX009501
*C. fructicola*	C1216.5	*Persea americana*	Australia	JX010166	JX009946	JX009529
*C. gloeosporioides*	CBS 273.51	*Citrus limon*	Italy	JX010148	JX010054	JX009558
*C. gloeosporioides*	CBS 100471	NA	Brazil	KC566719	KC566573	KC566865
*C. musae*	CBS 116870	*Musa* sp.	USA	JX010146	JX010050	JQ005840
*C. nupharicola*	C1275.26	*Nuphar lutea*	USA	JX010188	JX010031	JX009582
*C. siamense*	C1316.5	*Hymenocallis americana*	China	JX010278	JX010019	JX009441
*C. thailandicum*	MFUCC1101	*Hibiscus rosa-sinensis*	Thailand	JN050242	JN050231	JN050220
*C. theobromicola*	C1316.13	*T. cacao*	Panama	JX010294	JX010006	JX009444
*C. tropicale*	CBS 124946	*T. cacao*	Panama	KC566806	KC566660	KC566952
*Glomerella glycines*	ATCC 62257	*Glycine max*	USA	KC110794	KC110812	KC110830

^a^ Not available; ^b^ New Zealand; and bold indicates, sequenced and assigned accession numbers in the present study.

### 4.5. Morphological Observation

The isolates CNU122031 and CNU122032 were used for morphological description. Colony characteristics (color, size, and texture) were determined after 7 days at 25 °C in the dark grown on PDA plates. Conidial morphology was examined in standard conditions [13]. Plates were maintained in a chamber without humidity control at 25 °C and held under a fluorescent light/dark cycle of 12/12 h. Conidia were mounted in lactophenol picric acid solution (SIGMA-ALDRICH, Munich, Germany) and measured using a light microscope (OLYMPUS BX50, Tokyo, Japan) and an Artray Artcam 300MI digital camera system (Artray Co., Ltd., Tokyo, Japan). Randomly selected conidia were counted for the morphological description. A slide culture technique [35] was used for the production of appresoria and their size and shape were examined.

## 5. Conclusions

One of the main objectives of the present study was to find unreported fungal species in Korea. The *ITS* sequence-based identification showed that CNU122031 and CNU122032 were consistent with *C. fructicola* and *C. brevisporum*, respectively, both of which were previously unrecorded from Chinese borxthorn plant and *C. brevisporum* is new record in Korea. Further study was needed to clarify the identification. A multi-locus molecular study was carried out with Actin and GAPDH gene sequences. The combined dataset produced a clear molecular identification for these two fungi. Each fungus was isolated as endophytic fungi previously. *C. brevisporum* was reported as an endophytic new fungus from Thailand. The distinguishing characteristics of the present fungi and their reference species and related species was described. We conclude that Chinese boxthorn in Korea is the host species of unrecorded fungi and plays a potentially important role in understanding microbial diversity in Korea.
